# Salvianolic Acids: Potential Source of Natural Drugs for the Treatment of Fibrosis Disease and Cancer

**DOI:** 10.3389/fphar.2019.00097

**Published:** 2019-02-20

**Authors:** Lunkun Ma, Liling Tang, Qian Yi

**Affiliations:** ^1^Key Laboratory of Biorheological Science and Technology, Ministry of Education, College of Bioengineering, Chongqing University, Chongqing, China; ^2^Department of Physiology, School of Basic Medical Sciences, Southwest Medical University, Luzhou, China

**Keywords:** *Salvia miltiorrhiza*, compounds, fibroblasts, traditional medicine, epithelial-mesenchymal transition

## Abstract

Salvianolic acids, the most effective and abundant compounds extracted from *Salvia miltiorrhiza* (Danshen), are well known for its good anti-oxidative activity. Danshen has been extensively used as a traditional medicine to treat cardiovascular-related diseases in China and other Asian countries for hundreds of years. Recently, more and more studies have demonstrated that salvianolic acids also have a good effect on the alleviation of fibrosis disease and the treatment of cancer. *In vivo* and *in vitro* experiments have demonstrated that salvianolic acids can modulate signal transduction within fibroblasts and cancer cells. It is discovered that the cancer treatment of salvianolic acids is not only because salvianolic acids promote the apoptosis of cancer cells, but also due to the inhibition of cancer-associated epithelial-mesenchymal transition processes. In this article, we review a variety of studies focusing on the comprehensive roles of salvianolic acids in the treatment of fibrosis disease and cancer. These perspectives on the therapeutic potential of salvianolic acids highlight the importance of these compounds, which could be the novel and attractive drugs for fibrosis disease and cancer.

## Introduction

*Salvia miltiorrhiza* (Danshen) is one of the commonly used drugs in traditional Chinese medicine and has a long history of the clinical application. According to Chinese medicine records, *S. miltiorrhiza* can promote blood circulation and relieve congestion. Therefore, *S. miltiorrhiza* is widely used in patients suffered from cardiovascular diseases, hyperlipidemia, and acute ischemic stroke (Zhou et al., 2005; Wang et al., 2007). The composition of *S. miltiorrhiza* is complex and diverse. The active ingredients of *S. miltiorrhiza* are divided into water-soluble compounds and lipid-soluble compounds (Liu et al., 2007). Salvianolic acids are the most water-soluble compounds in *S. miltiorrhiza*. Among salvianolic acids, salvianolic acid A (Sal A) and salvianolic acid B (Sal B) are the most abundant components.

Fibrosis, a chronic stage of many diseases that affects millions of people of all racial and ages groups, is characterized by excessive deposition of extracellular matrix (ECM) and then leads to tissue structural damage and organ dysfunction (Zhang and Friedman, 2012; Krenkel and Tacke, 2017). Fibrosis is the end stage of chronic inflammatory responses caused by a variety of stimuli factors including chemical insults, autoimmune reactions, radiation, tissue injury, allergic responses, and persistent infections (Wynn, 2007). Fibrosis is the process that non-physiological scar formation which plays a crucial role in the destruction of the liver, kidney, heart, lung, and other parenchymal organs. More and more studies have shown that the development of cancer has an important relationship with fibrosis diseases. For instance, most lung cancer cases are found in the lung periphery and lower lobes, and changes in lung fibrosis also occur mainly in these areas (Nagai et al., 1992; Aubry et al., 2002). In addition, 80–90% of hepatocellular carcinomas (HCCs) develop toward cirrhosis or liver fibrosis (El-Serag, 2011). Although some great progress has been made to understanding the pathophysiological mechanism of fibrosis diseases, current therapeutic options are still limited to appear as effective anti-fibrosis agents. Currently, there are still no specific treatment drugs to prevent or reverse the fibrosis. Therefore, it is very urgent to find and develop anti-fibrosis drugs.

In the past few years, although the good therapeutic effect of salvianolic acids on cardiovascular protection and neural protection has been proved (Wang S. X. et al., 2010; Chen et al., 2011), the most important impacts of salvianolic acids are cancer treatment and alleviation of fibrosis diseases (Liu et al., 2010). The mechanism of how salvianolic acids regulate fibroblasts and cancer cells has been widely studied in recent years. In this article, we searched the references from relevant papers and PubMed databases. We showed an overview of the advances in illustrating the effects of salvianolic acids against fibrosis diseases and cancer. In addition, we classified the functional mechanisms and pharmacological activity of salvianolic acids in the treatment of fibrosis diseases and cancer. We also further summarized the therapeutic effects of salvianolic acids in animals (Table 1).

**Table 1:** *In vivo* experiment for evaluating the effects of Salvianolic acids.

Compound	Animal model	Effects	Reference	
Sal A Sal B	High-fat diet (HFD)-fed and streptozotocin (STZ)-induced diabetic rats	Reduced hepatocyte apoptosis and the expression of α-SMA and TGF-β1 in the liver	Qiang et al., 2014
	Bleomycin (BLM)-induced rats	Attenuated collagen deposition and alveolar wall thickness	Pan et al., 2014
	Monocrotaline (MCT)-induced pulmonary arterial hypertension (PAH) rats	Increased the expression of bone morphogenetic protein type II receptor (BMPRII) and phosphorylated Smad1/5	Chen Y. C. et al., 2016
	Male Sprague-Dawley rats myocardial infarct (MI) induced by ligation of left anterior descending coronary artery (LAD)	Up-regulated Nrf2 and inactivated the P2x7r-Pkr-Nlrp3 signaling pathway	Li et al., 2014; Mao et al., 2017
	Monocrotaline (MCT)-induced pulmonary arterial hypertension (PAH) model rats Xenograft mouse model	Potentiated the ischemia-induced neovascularization. Improved vascular function, decreased TGF-β1 level and inhibited inflammation	Chen et al., 2017
	Cardiac Remodeling in Spontaneously Hypertensive Rats	Inhibited fibroblast migration and the secretion of Cytokine such as ICAM, IL-6, and sVCAM-1	Jiang et al., 2013
	5/6 nephrectomized (5/6Nx) rats animal model	Inhibited the activation of NF-κB and p38 MAPK signaling pathways	Zhang et al., 2018
	Bilateral common carotid artery occlusion (BCCAO)-induced vascular dementia (VD) model rats	Suppressed acute myeloid leukemia (AML) tumor growth Ameliorated cognitive deficits in bilateral common carotid artery occlusion (BCCAO)-induced	Pei et al., 2018
		Vascular Dementia (VD) model rats	Ma X. et al., 2017
	Bleomycin-instilled mouse model of pulmonary fibrosis	Inhibited inflammatory cell infiltration, alveolar structure disruption, and collagen deposition	Liu Q. et al., 2016
	Renal interstitial fibrosis (RIF) was induced in rats by oral administration of HgCl_2_	Decreased the expression of α-SMA, TGF-β1, TbetaR-I, p-Smad2/3 and MMP-2 but increased the expression of E-cadherin	Wang Q. L. et al., 2010
	CCl4-treated mice model	Suppressed the activation of HSCs, leading to inhibition of cell proliferation, type I collagen and alpha-smooth muscle actin	Yu et al., 2015
	7,12-dimethylbenz[a]anthracene (DMBA)-induced oral carcinogenesis in hamsters	Inhibited angiogenesis and decreased the expression of hypoxia-inducible factor 1α (HIF-1α) and VEGF	Zhou et al., 2006
	Nude mice with HNSCC solid tumor xenografts DMBA-induced hamster oral carcinogenesis	Inhibited the growth of squamous cell carcinoma of the head and neck through cyclooxygenase-2 and the apoptotic pathway	Hao et al., 2009
		Modulated aberrant glucose metabolism via the PI3K/AKT/HIF-1 alpha signaling pathways, attenuated DMBA-induced metabolic perturbation	Wei et al., 2012, 2018


## Salvianolic Acids

**FIGURE 1:**
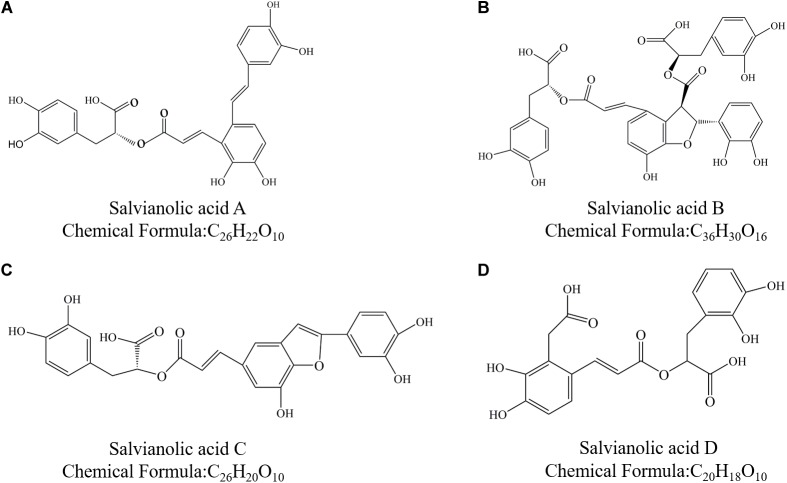
Chemical structure of Salvianolic acids. **(A)** Salvianolic acid A; **(B)** Salvianolic acid B; **(C)** Salvianolic acid C; and **(D)** Salvianolic acid D.

To date, there are more than 10 different salvianolic acids been identified and named: salvianolic acid A, B, C, D, E, F, G, etc. Sal A and Sal B are the most abundant compounds among salvianolic acids. Danshensu [(R)-3- (3, 4-Dihydroxyphenyl)-2-hydroxypropanoic acid] is the basic chemical structure of various salvianolic acids (Chen et al., 2014; Du et al., 2016). Salvianolic acid A is formed by a molecule Danshensu and two molecules caffeic acid (Xu et al., 2014). Salvianolic acid B is formed by three molecules Danshensu and a molecule caffeic acid. Salvianolic acid C is a combination of two molecules Danshensu (Tang et al., 2016). Salvianolic acid D is a dimer of caffeic acid. As is shown in Figure 1, the structure of these four salvianolic acids all contains a phenolic acid structure.

Salvianolic acids, the compounds found in danshen, have well-established bioactivities. Both *in vitro* and vivo, most of the salvianolic acids showed anti-inflammatory, antioxidative and the activities of free radical scavenging, and could protect cells from a variety of harmful factors (Zhao et al., 2008). Salvianolic acids have been used in traditional Chinese medicine for the treatment of cardiovascular diseases for more than a thousand years (Li et al., 2007). Compared with other phenolic compounds in danshen, salvianolic acids have more stronger antioxidant activity and other biological activities (Du et al., 2016). Although salvianolic acids could scavenge free radicals directly, they may not be existed in the body at the high concentrations. The antioxidant activity of salvianolic acids may lead to the increase in the expression of antioxidant enzymes and the decrease of the expression of pro-antioxidant enzymes, such as the activation of Nrf2/HO-1 signaling (Zhang et al., 2014).

## Effects of Salvianolic Acids on Fibrosis Disease

Recent studies show that salvianolic acids have good effects on some chronic fibrosis disease, especially on liver fibrosis and pulmonary fibrosis. We summarized the functional role of salvianolic acids in the fibrosis of several organs and its potential as a novel therapeutic target (Table 2).

**Table 2:** The effects of Salvianolic acids on fibrosis related diseases.

Type of fibrosis disease	Compounds	Effects	Reference
Liver fibrosis	Sal A	Inhibited the activities of AlaAT and AspAT in serum, decreased the content of Hyd and MDA; reduced type I and type III collagen	Hu et al., 1997, 2000; Liu et al., 2000
		Promoted the apoptosis of HSCs and inhibited the activation and proliferation of HSCs	Liu et al., 2000; Lin et al., 2006a
		Decreased Bcl-2 protein, Cyclin D1 protein, Cyclin E protein, and p- AKT; proteins p21; p27 and caspase-3	Lin et al., 2006a
		Suppressed the activity of ALT and MDA	Hu et al., 2000; Liu et al., 2001
		Decreased α-SMA and TGF-β1 expression and reduced hepatocyte apoptosis	Qiang et al., 2014
		Alleviated BDL- and PDGF-BB-induced liver injury and ER stress through SIRT1-mediated HSF1 deacetylation	Zhu et al., 2018
	Sal B	Inhibited LX-2 cells proliferation and decreasedα-SMA expression	Hou et al., 2011
		Blocking H_2_O_2_-induced mitochondrial deformation and dysfunction	Liu et al., 2017
		Inhibited the activation of HSCs and decreased the expression of type I collagen and α-SMA protein by the lincRNA-p21-mediated Wnt/β-catenin pathway	Yu et al., 2015, 2016b, 2017
		Inhibits CCL4-induced liver fibrosis by the NF-Kb/IκBα signaling pathway	Wang et al., 2012
		Down-regulates Ang II signaling	Li et al., 2012
		Inhibited the activation of Rho A and ROCK II and the downstream MYPT1 phosphorylation at Thr (696)	Xu et al., 2012
Pulmonary fibrosis	Sal A	Reduced alveolar wall thickness and collagen deposition	Pan et al., 2014
		Affecting the expression of cycle-associated proteins (cyclin D1, cyclin E1, and cyclin B1) and apoptosis-related proteins (Bcl-2 and caspase-3)	Pan et al., 2014; Chen Y. C. et al., 2016
		Activated the BMPRII-Smad pathway and inhibiting apoptosis	Chen Y. C. et al., 2016
Renal fibrosis Cardiac fibrosis	Sal B	Reduced lung hydroxyproline content and lung type I collagen expression	Liu et al., 2015
		Inhibited Smad-dependent signaling and the Smad-independent MAPK pathway	Liu Q. et al., 2016
		Decreased expression of ROS-producing enzyme Nox4	Liu B. et al., 2016
	Sal A and Sal C	Attenuated the expression of human chemokine ligand 5 (CCL5) and chemokine ligand 10 (CXCL10)	Li et al., 2015
	Sal B	Modulating the PI3K/AKT signaling pathway	Ma Z. G. et al., 2017
		Inhibited the expression of α-SMA protein and maintaining epithelial phenotype	Zhou et al., 2009; Lu et al., 2010
	Sal A	Inhibited the expression of matrix metalloproteinase-9 (MMP-9)	Jiang et al., 2013
	Sal B	Attenuated Ang II-induced myocardial fibrosis by inhibiting the NF-κB pathway	Wang et al., 2018


### Salvianolic Acids and Liver Fibrosis

Liver fibrosis is a common final stage of several chronic liver diseases and is characterized by excessive deposition of ECM and collagen in response to liver injury. Many liver diseases, such as liver disease caused by drug damage, alcoholic liver disease, viral hepatitis, metabolic liver disease induced by excessive metal ionization, autoimmune liver disease and certain congenital diseases caused by liver damage, can be expected to result in liver fibrosis. Activation of hepatic stellate cells (HSCs) and their differentiation into myofibroblasts are deemed to be a critical step in the development of liver fibrosis (Friedman, 2003; Lotersztajn et al., 2005). Activation, chemotaxis and proliferation of HSCs, secretion of profibrotic cytokines and increased collagen synthesis can lead to imbalance of ECM accumulation and degradation, further causing abnormal deposition of liver fiber connective tissue, eventually leading to liver fibrosis, liver structural damage and abnormal liver function.

**FIGURE 2:**
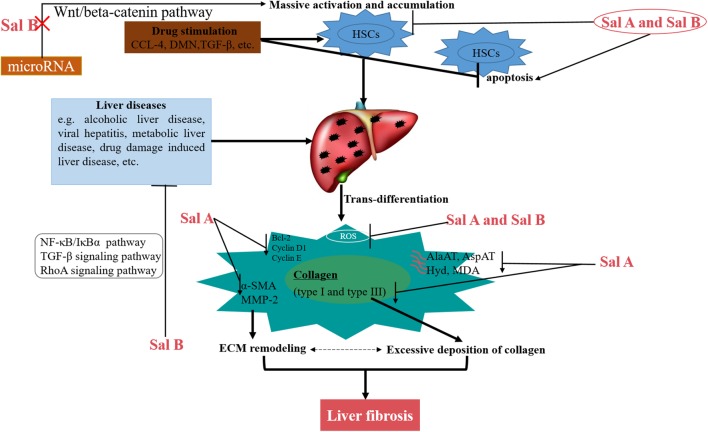
Schematic model of the multiple mechanisms of Salvianolic acids for liver fibrosis treatment.

Numerous *in vivo* and *in vitro* experiments indicate the effectiveness of salvianolic acids for improving liver fibrosis (Figure 2). The inhibition of liver fibrosis by Sal A may relate to its anti-lipid peroxidation. As early as more than a decade ago, researchers have confirmed that Sal A inhibited the activities of aminotransferase (AlaAT) and aspartate aminotransferase (AspAT) in serum, decreased the content of hydroxyproline (Hyd) and malondialdehyde (MDA), alleviated live fibrosis, and reduced the deposition of type I and type III collagen in the liver matrix (Hu et al., 1997, 2000; Liu et al., 2000). In addition, Sal A also promoted the apoptosis of HSCs and inhibited the activation and proliferation of HSCs (Liu et al., 2000, Lin et al., 2006a). Sal A decreased the expression of Bcl-2 protein, Cyclin D1 protein and Cyclin E protein in HSCs cells, induced the expression of cyclic inhibitory proteins p21 and p27, inhibited the phosphorylation of AKT and PDGF, and enhanced the activity of caspase-3 (Lin et al., 2006a). Besides, the activity of alanine aminotransferase (ALT) in serum and the activity of MDA can be suppressed by Sal A (Hu et al., 2000; Liu et al., 2001). In general, patients with type 2 diabetes have an increased risk of developing liver fibrosis. Qiang et al. (2014) reported that Sal A prevented the pathological progression of liver fibrosis in streptozotocin (STZ)-induced diabetic rats. Sal A significantly reduced hepatocyte apoptosis and down-regulated the expression of α-smooth muscle actin (α-SMA) and transforming growth factor β1 (TGF-β1) in the liver (Qiang et al., 2014). The underlying mechanism may be linked to the reduction of oxidative stress and protection of mitochondria. In addition to Sal A, Sal B can also alleviate liver fibrosis (Li et al., 2012). Sal B inhibited the proliferation of LX-2 cells and down-regulated the expression of α-SMA protein (Hou et al., 2011). The study also found that Sal B protected the liver from damage by effectively blocking H_2_O_2_-induced mitochondrial deformation and dysfunction in the human liver cell line HL7702 (Liu et al., 2017).

The enhancement of oxidative stress is closely related to liver fibrosis (Sanchez-Valle et al., 2012; Domitrovic et al., 2013). Sal A and Sal B attenuated PDGF-induced ROS formation in HSCs through different signaling pathways (Tsai et al., 2011). The inhibition of oxidative stress by Sal A and Sal B may be through the inhibition of nicotinamide adenine dinucleotide phosphate (NADPH) oxidase (Tsai et al., 2010). Many other experiments have also demonstrated that Sal A and Sal B eliminated the accumulation of ROS, alleviated oxidative damage, and attenuated HSCs activation in hepatocytes (Lin et al., 2006b; He et al., 2010; Tsai et al., 2010). Zhu et al. (2018) demonstrated that Sal A treatment alleviated BDL- and PDGF-BB-induced liver injury and ER stress through SIRT1-mediated HSF1 deacetylation recently.

It is well known that aberrant activating of Wnt/beta-catenin pathway can accelerate liver fibrosis development (Yu et al., 2016a). It has been reported that microRNA (miRNA)-mediated Wnt/β-catenin is involved in the activation of HSCs during liver fibrosis (Li G. et al., 2016; Yu et al., 2016c). Sal B can alleviate liver fibrosis by inhibiting the activation of HSCs and decreasing the expression of type I collagen and α-SMA protein by the lincRNA-p21-mediated Wnt/β-catenin pathway (Yu et al., 2015, 2016b, 2017). Sal B also inhibited CCL4-induced liver fibrosis by the NF-Kb/IκBα signaling pathway (Wang et al., 2012). Besides, Sal B prevented the activation of HSCs by influencing the TGF-β signaling pathway in a rat model of liver fibrosis induced by intraperitoneal injection of dimethylnitrosamine (DMN) (Tao et al., 2013). Numerous *in vivo* and *in vitro* experiments have also demonstrated that Sal B attenuated liver fibrosis by affecting other signaling cascades. For example, Sal B attenuated ET-1-induced HSCs contraction by inhibiting the activation of Rho A and ROCK II and the downstream MYPT1 phosphorylation at Thr (696) (Xu et al., 2012), and this process is associated with inhibition of the Rho A signaling pathway. In LX-2 cells, Sal B can inhibit the expression of Col I independent of TGF-β1 stimulation, which is associated with direct inhibition of p38 signaling and inhibition of crosstalk between Smad and ERK signaling (Lv and Xu, 2012). When Sal B exerts its anti-liver fibrosis, it also down-regulated Ang II signaling during HSCs activation (Li et al., 2012).

Although a large number of experiments have proved that the salvianolic acids have a good treatment effect on liver fibrosis, there are still many proved shortcomings. For instance, experiment shows that Sal B may induce liver fibrosis in rats by down-regulating CD14 expression and blocking endotoxin signaling to antagonize CCL4 (Liu et al., 2011). Therefore, it is very necessary to verify the effect of salvianolic acids on liver fibrosis.

### Salvianolic Acids and Pulmonary Fibrosis

**FIGURE 3:**
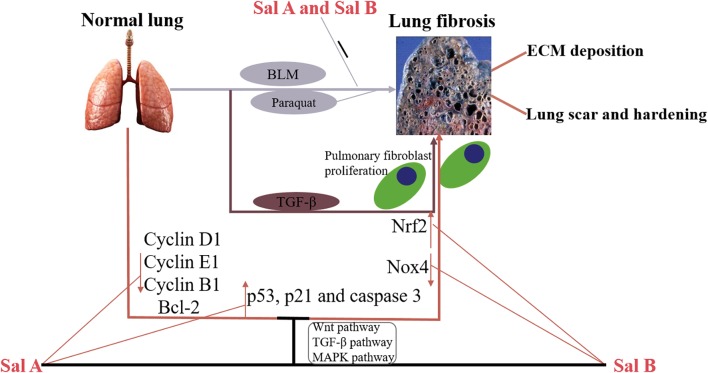
Schematic model of the multiple mechanisms of Salvianolic acids for pulmonary fibrosis treatment.

Pulmonary fibrosis involves a series of heterogeneous lung diseases characterized by abnormally excessive accumulation of ECM leading to scarring and sclerosis of the lung tissue, loss of alveolar structure, ultimately leading to gas exchange and fatal respiratory failure (Strieter and Mehrad, 2009; Noble et al., 2012). Idiopathic pulmonary fibrosis (IPF) is the most common form of pulmonary fibrosis, which poses a serious threat to human health. Its pathogenesis is unknown, and its life expectancy after diagnosis is very short (Thannickal et al., 2014). To date, despite decades of extensive research on IPF, there is still a lack of effective treatment. Therefore, finding safe and effective drugs for the treatment of pulmonary fibrosis is essential (Thannickal, 2013; Wolters et al., 2014). As is shown in Figure 3, we summarized the multiple mechanisms of salvianolic acids for pulmonary fibrosis treatment.

Pan et al. (2014) found *in vivo* that Sal A attenuated BLM-induced pulmonary fibrosis in rats, and reduced alveolar wall thickness and collagen deposition. Besides, *in vitro* experiments have demonstrated that Sal A significantly inhibited fibroblast proliferation, adhesion and migration by affecting the expression of cycle-associated proteins (cyclin D1, cyclin E1, and cyclin B1) and apoptosis-related proteins (Bcl-2 and caspase-3) (Pan et al., 2014; Chen Y. C. et al., 2016). Sal B also effectively inhibited BLM-induced pulmonary fibrosis in mice by reducing the mouse lung interstitial collagen fiber positive region model, reducing lung fibroblast number, and decreasing lung Hyd content and lung type I collagen expression (Liu et al., 2015).

Inhibition of pulmonary fibrosis by salvianolic acids may be associated with the Smad signaling pathway. For example, Sal A ameliorated pulmonary remodeling in monocrotaline (MCT)-induced pulmonary arterial hypertension (PAH) rats by activating the BMPRII-Smad pathway and inhibiting apoptosis (Chen Y. C. et al., 2016). Liu Q. et al. (2016) demonstrated that Sal B attenuated pulmonary fibrosis by inhibiting Smad-dependent signaling and the Smad-independent MAPK pathway. Sal B inhibited the expression of TGF-β1 and phosphorylation of Smad3 in a rat model of pulmonary fibrosis induced by paraquat. In addition, Sal B enhanced nuclear translocation and expression of nuclear factor erythrocyte 2-related factor 2 (Nrf2) and decreased the expression of ROS-producing enzyme Nox4 (Liu B. et al., 2016).

### The Effects of Salvianolic Acids on Other Fibrosis Disease

Renal fibrosis is an inevitable process in the development of all chronic kidney diseases, eventually leading to renal failure. Renal fibrosis is characterized by accumulation of fibroblasts and excess matrix proteins (Eddy and Neilson, 2006). Numerous studies have demonstrated that renal fibrosis is mediated by a variety of mediators through a variety of mechanisms and pathways, including growth factors, cytokines, and metabolic toxin stress molecules. Among them, TGF-β1 has been taken into account as an important mediator in the pathogenesis of renal fibrosis (Wang et al., 2005; Bottinger, 2007). Studies have shown that both Sal A and Sal B can be involved in the regulation of TGF-β1-induced renal fibrosis. Ma et al. (2016) demonstrated that Sal A inhibited renal fibrosis by inhibiting the TGF-β1/Smad signaling pathway. Sal B can inhibit the fibrosis process of HK-2 cells by inducing the expression of miR-106b-25 (Tang et al., 2014). Similar studies have shown that Sal B does have a good relief effect on TGF-β1-induced renal fibrosis (Pan et al., 2011; Li et al., 2017). Studies also have shown that Sal A and Sal C inhibited renal fibrosis by attenuating the expression of human chemokine ligand 5 (CCL5) and chemokine ligand 10 (CXCL10) (Li et al., 2015). Besides, Sal B reduced inflammation and oxidative stress by modulating the PI3K/AKT signaling pathway (Ma Z. G. et al., 2017). When the experiment was supplemented with additional Sal B, the expression of PI3K protein and the ratio of p-AKT/AKT was significantly up-regulated. Sal B also has a beneficial effect on renal fibrosis by inhibiting the expression of α-SMA protein and maintaining epithelial phenotype (Zhou et al., 2009; Lu et al., 2010). Recent studies have also found that Sal A attenuated kidney damage in rats by inhibiting the activation of NF-κB and p38 MAPK signaling pathways (Zhang et al., 2018). Therefore, it is significant to further investigate the roles of salvianolic acids in renal fibrosis, which may have therapeutic potential for progressive renal fibrosis.

Cardiac fibrosis is a common pathological condition. Almost all major heart diseases, including hypertension, cardiomyopathy, and myocardial infarction, can lead to myocardial remodeling characterized by excessive deposition of ECM proteins secreted by myofibroblast, further leading to decreased myocardial compliance, cardiac dysfunction and even heart failure (Leask, 2015; Travers et al., 2016). Jiang et al. (2013) found Sal A inhibited fibroblast migration and the secretion of Cytokine such as intercellular adhesion molecule (ICAM), interleukin-6 (IL-6) and soluble vascular cell adhesion molecule-1 (sVCAM-1). These effects are achieved by competitively inhibiting the expression of matrix metalloproteinase-9 (MMP-9). Likewise, Sal B selectively inhibited MMP-9 activities to prevent fibrosis without affecting MMP-9 expression (Jiang et al., 2010). Recently, Wang et al. (2018) found that Sal B attenuated Ang II-induced myocardial fibrosis by inhibiting the NF-κB pathway. In this process, Sal B reduced the nuclear translocation of the NF-κB p65 subunit. It is highly desirable to further develop the potential clinical application of salvianolic acids to protect the heart.

## Potential Applications of Salvianolic Acids for the Treatment of Cancer

Cancer is a serious threat to human health. It is extremely urgent to develop drugs that safely and effectively treat cancer and to find the corresponding targets for cancer treatment. It is worth noting that many traditional Chinese medicines have achieved good results in the treatment of cancer. Among them, a large number of studies have shown that salvianolic acids have a good effect in treating various types of cancer (Table 3).

**Table 3:** The effects of Salvianolic acids on cancer.

Type of cancer	Compounds	Cells/Tissues	Effects	Reference
Breast cancer	Sal A	MCF-7/PTX cells	ABC transporter Drug resistance PI3K/Akt signaling Transgelin 2 Cell migration and invasion	Cai et al., 2014; Zheng et al., 2015
		MCF-7 cells	Cell proliferation Cell apoptosis Multidrug resistance	Wang et al., 2015
			Mitochondrial membrane potential	
		MCF7cells MDA-MB-231 cells	Multidrug resistance Drug delivery system	Ding et al., 2016
Head and neck squamous cell carcinoma	Sal B	HN13 cells HN30 cells	Cell apoptosis Cell cycle	Li H. et al., 2016
		JHU-022 cells JHU-013 cells	Cell growth Tumor volumes	Hao et al., 2009
		JHU-06 cells JHU-011 cells JHU-013 cells JHU-022 cells	Cell apoptosis Tumor volumes	Zhao et al., 2010
Lung cancer	Sal A	W1-38 cells A549 cells	P46 (JNK/SAPK) expression	Li et al., 2002
	Sal B	A549 cells	COX-2 activity Cell growth	Tao et al., 2014
	Salvianolic acids	A549 cells	PTEN/PI3K/AKT pathway EMT	Yang et al., 2017
	Sal A	A549 cells	MDR1 microRNA	Chen F. Y. et al., 2016
Squamous cell carcinoma	Sal B	Hamsters tissues	Angiogenesis Hypoxia-inducible factor 1alpha Vascular endothelium growth factor protein	Zhou et al., 2006
Oral squamous cell carcinoma	Sal B	CAL27 cells SCC4 cells	HIF-1α, TNFα MMP9 THBS2 anti-angiogenic	Yang et al., 2011
	Sal A	SCC-9 cells SCC-25 cells	MMP-2 c-Raf/MEK/ERK Invasion and migration of OSCC	Fang et al., 2018
Retinoblastoma	Sal B	HXO-RB44 cells	Cell apoptosis Cell cycle Cell growth	Liu, 2012
Ovarian cancer	Sal B	SKOV3 cells	Cell apoptosis livin Cell growth	Yan, 2016
Melanoma	Danshensu	B16F10 cells	Tumor angiogenesis Tumor invasion	Zhang et al., 2010
Colorectal cancer	Sal B	LoVo cells HCT-116 cells	CD44, SOX2 ABCG2 multidrug resistance	Guo et al., 2018
		HCT116 cells HT29 cells	AKT/mTOR pathway Cell autophagy	Jing et al., 2016
Liver cancer	Sal B	HCC cells	Cell apoptosis Cell autophagy AKT/mTOR pathway	Gong et al., 2016
	Salvianolic acids	HepG2	Cell invasion TGF-β/ Smad	Liu et al., 2010
	Sal A	H22 cells	Tumor growth	Li et al., 2002


### Salvianolic Acids for the Treatment of Breast Cancer

Some studies have predicted that Sal A and Sal B have a good therapeutic effect on breast cancer. MCF-7/PTX cells have strong migration and invasion abilities and are highly resistant to the anticancer drug paclitaxel. Cai et al. (2014) and Zheng et al. (2015) found that Sal A reversed the resistance of ccc cells to paclitaxel. Transgelin 2 inhibited the apoptosis of MCF-7/PTX cells by activating phosphatidylinositol 3-kinase (PI3K)/Akt signaling pathway and mediating the drug resistance of paclitaxel in breast cancer patients. In this process, Sal A suppressed transgelin 2 expression by inhibiting PI3K/Akt pathway activation and adenosine triphosphate binding cassette (ABC) transporter (Cai et al., 2014). At the same time, the metastatic and invasive ability of MCF-7/PTX cells was also significantly inhibited by Sal A (Zheng et al., 2015). Sal A is considered a potential drug to against multidrug resistance (MDR), the main reason for incurable breast cancer. Wang et al. (2015) demonstrated that Sal A induced apoptosis in MCF-7 cells by increasing the activity of caspase-3 and Bax, disrupted the membrane potential of mitochondria, and down-regulated the expression of Bcl-2.

In addition, Sal B also inhibited the proliferation of breast cancer cells and promoted their apoptosis (Sha et al., 2009, 2011). Ding et al. (2016) demonstrated that the FA-PEG-TiO2 nanoparticles loaded with curcumin and Sal B has a good prospect for the treatment of breast cancer by optimizing the drug delivery system.

### Salvianolic Acids for the Treatment of Squamous Cell Carcinoma

In recent years, studies have also focused on the effects of salvianolic acids on squamous cell carcinoma (SCC). Zhou et al. (2006) first discovered that Sal B reduced the incidence of SCC by inhibiting angiogenesis and decreasing the expression of hypoxia-inducible factor 1α (HIF-1α) and VEGF. *In vivo* and *in vitro* experiments further demonstrated that Sal B inhibited the growth of SCC of the head and neck through cyclooxygenase-2 and the apoptotic pathway (Hao et al., 2009; Zhao et al., 2009, 2010). Overexpression of cyclooxygenase-2 (COX-2) is associated with an increased risk of head and neck cancer. Studies have found that Sal B inhibited the growth of HNSCCs and reduced the formation of solid tumors by reducing the expression of COX-2 (Hao et al., 2009; Zhao et al., 2010). Li H. et al. (2016) found that nano-SalB arrested the cell cycle of head and neck squamous cell carcinoma cells (HNSCCs) and induced apoptosis. In addition, Sal B and low-dose celecoxib (a selective inhibitor of COX-2) work better for the treatment of head and neck SCC (Zhao et al., 2010). Sal B has a certain therapeutic effect on 7, 12-dimethylbenz[a]anthracene (DMBA)-induced oral cancer. Yang et al. (2011) also demonstrated that Sal B inhibited the growth and angiogenesis of oral cancer cells by down-regulating the expression of HIF-1α, TNFα and MMP9 genes and up-regulating the expression of THBS2. Wei et al. (2012, 2018) found that Sal B inhibited the glycolysis of oral SCC by targeting PI3K/AKT/HIF-1α signaling pathway and attenuated DMBA-induced metabolic disorders. Recently, Fang et al. (2018) found that Sal A inhibited the metastasis of oral SCC by inhibiting the c-Raf/MEK/ERK pathway controlling the expression of matrix metallopoteinase-2 (MMP-2).

### Salvianolic Acids for the Treatment of Lung Cancer and Liver Cancer

Salvianolic acids also have a good effect in the treatment of lung cancer and liver cancer. Li et al. (2002) found that Sal A inhibited the growth of mouse lung cancer cells by inhibiting the expression of c-myc and P46 (JNK/SAPK). *In vivo* experiments have also demonstrated that Sal B has a certain inhibitory effect on lung cancer (Tao et al., 2014). Interaction of salvianolic acids with other compounds inhibited the migration and invasion of A549 cells, and inhibited the epithelial-mesenchymal transition (EMT) process of A549 cells through the PTEN/PI3K/AKT pathway (Yang et al., 2017). In addition, Chen F. Y. et al. (2016) also found that Sal A inhibited the expression of the MDR gene MDR1 in lung cancer through miRNA expression and regulation of target genes. Gong et al. (2016) found that Sal B induced apoptosis of human hepatocellular carcinoma cells (HCC) through the mitochondrial apoptosis pathway. Interestingly, Sal B induced autophagy in both hepatoma cells and colorectal cancer cell lines (Gong et al., 2016; Jing et al., 2016). Sal B-induced autophagy may play a pro-apoptotic role, and the AKT/mTOR signaling pathway may be involved in the Sal B induced autophagy process (Gong et al., 2016).

There are also studies on the effects of salvianolic acids on other cancers. For example, Liu (2012) found that Sal B inhibited the growth and promoted apoptosis of retinoblastoma HXO-RB44 cells by up-regulating Caspase-3 expression and inducing cell cycle arrest. Yan (2016) found that Sal B inhibited the growth of ovarian cancer cell line SKOV3 and promoted its apoptosis by up-regulating the expression of Caspase-3, down-regulating the expression of livin, and blocking the cell cycle. In addition, Danshensu, an active component of salvianolic acids, inhibited the development of melanoma by affecting tumor angiogenesis and tumor invasion (Zhang et al., 2010). Guo et al. (2018) demonstrated that Sal B reversed tumor MDR by down-regulating the expression of CD44, SOX2 and ABCG2 proteins in LoVo and HCT-116 colonic CSC xenografts. A recent study by Wu et al. (2018) indicated that *S. miltiorrhiza* extract inhibited hepatocarcinogenesis by modulating TGF-β/TβR and Imp7/8 protein expression, suggesting that *S. miltiorrhiza* has multiple targets in HCC treatment.

### Inhibition of Cancer-Associated Epithelial-Mesenchymal Transition by Sal B

Epithelial-mesenchymal transition is an important cellular program involved in cancer development. It is characterized by the ability of epithelial cells to transform into mesenchymal cells and gain migration and invasion (Kalluri and Weinberg, 2009; Yilmaz and Christofori, 2009). When cells undergo an EMT process, the expression levels of proteins that are in contact with each other such as E-cadherin and γ-catenin is decreased, and the expression of mesenchymal markers such as vimentin, N-cadherin and fibronectin is increased. The main signaling pathway regulating EMT is TGF-β, Notch and Wnt signaling pathways (Nelson and Nusse, 2004; Katsuno et al., 2013). Studies over the past few years support a significant role of EMT in accelerating cancer metastasis, and new treatments targeting the residual EMT-driven cancer cells in combination with conventional treatments can decrease drug resistance and metastasis formation (Yeung and Yang, 2017). Thus, it can be seen that effective inhibition of EMT plays an important role in the treatment of cancer metastasis.

**FIGURE 4:**
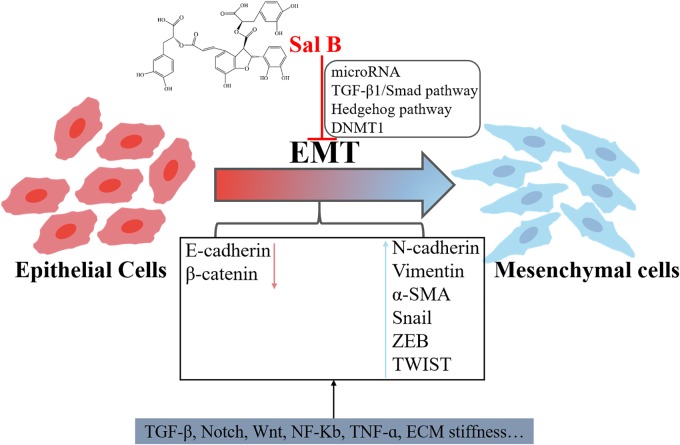
Inhibition of Salvianolic acid B on EMT process.

Recent studies have shown that Sal B inhibited the EMT process (Figure 4). Sal B can further inhibit the EMT process by regulating the levels of microRNAs. Yu et al. (2015) found that Sal B ameliorated liver fibrosis by inhibiting EMT and Hedgehog pathway, in which Sal B causes an increase in miR-152 and further upregulates the negative regulators of Hedgehog pathway (Patched1) and DNA Methyltransferase1 (DNMT1). In general, all three members of the miR-106b-25 cluster (miR-106b, miR-93, and miR-25) were significantly down-regulated during TGF-β1-induced EMT. However, Tang et al. (2014) found that Sal B treatment increased the miR-106b-25 cluster in a dose-dependent manner in HK-2 cells. MiR-106b attenuates EMT by reducing the expression of α-smooth muscle actin (α-SMA) and increasing the expression of E-cadherin. In addition, *in vitro* experiments also found that Sal B inhibited TGF-β1-induced EMT in HK-2 cells, a process involved in TGF-β/Smad signaling pathway (Yao et al., 2009; Wang Q. L. et al., 2010; Zhou et al., 2010). Another *in vivo* experiment by Wang Q. L. et al. (2010) demonstrated that Sal B inhibited HgCl_2_-induced kidney fibrosis in rats by decreasing the expression of α-SMA, TGF-β1, TbetaR-I, p-Smad2/3, and MMP-2 but increasing the expression of E-cadherin. Li et al. (2017) found that the nano-complex HCA-Chi-Ca-Sal B reversed TGF-β1-induced EMT in HK-2 cells. In addition, Yang et al. (2017) found that the complex formed by *S. miltiorrhiza* and other compounds inhibited EMT through the PTEN/PI3K/AKT signaling pathway.

These studies indicate that salvianolic acids have an inhibitory effect on EMT and can be used to treat cancer and fibrosis diseases. The reversal of EMT may have the consequence of promoting the regeneration of already disseminated cancer cells (Brabletz, 2012). Based on the understanding of cell plasticity, more *in vitro* and *in vivo* experiments should be conducted to study the combined effects of salvianolic acids for anti-EMT therapy.

## Conclusion and Future Perspective

Naturally derived drugs are an important source of novel medicines. Salvianolic acids containing polyphenol structure is effective antioxidants. Salvianolic acids reduce intracellular and intravascular oxidative stress, which protect cells from peroxidation, and free radical damage. Numerous experimental data indicated that salvianolic acids slowed the progression of fibrosis diseases by reducing excessive deposition of ECM. At the same time, a number of studies have shown that salvianolic acids also have potential anticancer effects by inhibiting the EMT process and cancer-related signal transduction. However, some issues still need to be taken seriously. First, the safety of salvianolic acids on the human body should be evaluated more carefully. Although salvianolic acids are a natural compound extracted from *S. miltiorrhiza*, their potential toxic derivatives cannot be ignored. A lot of studies with cells and animal models must be established to study the anti-fibrotic and anti-cancer effects of salvianolic acids. Different animal models are needed to comprehensively study the most suitable concentrations and doses of salvianolic acids. Secondly, phenolic hydroxyl groups in salvianolic acids have antioxidant activity and are prone to oxidation, so more attention should be paid to the stability of these compounds. It is necessary to use different animal models to assess the biological activity of salvianolic acids in the body. Finally, although salvianolic acids are water-soluble compounds and have high water solubility, their bioavailability can be further improved. It requires more *in vivo* and *in vitro* experiments to increase the bioavailability of salvianolic acids.

Taken together, salvianolic acids are a valuable class of natural compounds with potential for the treatment of fibrosis diseases and cancer. The anti-fibrotic and anti-cancer effects of salvianolic acids are mediated through a variety of molecular mechanisms. Such particular property makes salvianolic acids exceptional choices for future anticancer and anti-fibrotic disease drugs development. It is worth mentioning that the Chinese Food and Drug Administration (SFDA) has approved salvianolic acids for the treatment of chronic angina. Currently, it is widely used in clinical practice due to its good efficacy and safety. Other potential roles and potential mechanisms of salvianolic acids are at present being studied in order to be better applied to the treatment of other diseases.

## Author Contributions

All authors contributed for the preparation and read and approved the final manuscript. LM and LT were responsible for confirming the topic. LM were responsible for writing the first draft of this article. LT and QY contributed to furtherly editing and polishing the manuscript.

## Conflict of Interest Statement

The authors declare that the research was conducted in the absence of any commercial or financial relationships that could be construed as a potential conflict of interest.
